# Ethical Decision-Making in Indigenous Financial Services: QSuper Case Study

**DOI:** 10.1007/s10551-022-05253-4

**Published:** 2022-09-21

**Authors:** Clare J. M. Burns, Luke Houghton, Deborah Delaney, Cindy Shannon

**Affiliations:** Griffith University, Nathan, Australia

**Keywords:** Indigenous, Finance, Ethical decision-making, Moral awareness, Moral capacity, Storytelling, Empathy, Inclusive practice-financial inclusion

## Abstract

This case study details how and why integrating storytelling, empathy, and inclusive practice shifted QSuper, a large Australian finance organisation, from minimal awareness to moral awareness then moral capability in the delivery of services to Indigenous customers. During the Royal Commission into Misconduct in the Banking, Superannuation, and Financial Services Industry, QSuper were recognised for their exemplary service with Indigenous customers (Hayne, Interim report: Royal commission into misconduct in the banking, superannuation and financial services industry, Volume 1. Commonwealth of Australia, 2018; *Transcript of Proceedings, 13 August*, Commonwealth of Australia, 2018). This position was in stark contrast to the inaccessible service offerings of other financial organisations where some used predatory practices to sell unethical financial products to Indigenous Australians (Hayne, Interim report: Royal commission into misconduct in the banking, superannuation and financial services industry, Volume 1. Commonwealth of Australia, 2018; Hayne, Final report: Royal commission into misconduct in the banking, superannuation and financial services industry, Volume 1. Commonwealth of Australia, 2019a). Storytelling garned from visiting customers in remote communities and other meaningful activities involving inclusive practice to facilitate ethical decision-making in finance is different to standard functionalist finance approaches (Schinckus, Int Rev Financ Anal 40:103–106, 2015). Two empathetic questions asked within QSuper complementing the storytelling, were: “What is the right thing to do by the customer?” and “How would I feel if this were my mother?” Exploration into the lived reality of moral capacity is important based on the Commission finding many of the 490,000 finance staff do not know how to provide ethical services to vulnerable customers, in particular remote Indigenous customers (Australian Bureau of Statistics. Labour force, Australia, detailed. ABS. Retrieved from https://www.abs.gov.au/statistics/labour/employment-and-unemployment/labour-force-australia-detailed/latest-release, 2021; Hayne, Final report: Royal commission into misconduct in the banking, superannuation and financial services industry, Volume 1. Commonwealth of Australia, 2019a). Furthermore, there is minimal literature on the role of Indigenous storytelling to heighten moral awareness in the finance industry which was found to lead to better ethical outcomes.

## Introduction

Despite the Royal Commission into Misconduct in the Banking, Superannuation, and Financial Services Industry (Commission) and numerous inquiries dating back to 1893 ethical decision-making (EDM) in the Australian finance industry remains in question (Hayne, 2019a; Hickson & Turner, 2002). The Commission found Indigenous Australians were vulnerable due to low levels of financial literacy, a lack of access to financial services, remote geographical locations, non-conventional forms of personal identification, and language barriers (Hayne, 2018). Evidence was presented of finance products being sold to people in remote communities which they could not use, pay day lenders targeting Indigenous teenagers leaving them indebted before 20 years of age, and inaccessible superannuation (“super,” sometimes referred to as “retirement savings”) practices described as “stolen wages” (Joyner, 2019; *Transcript of Proceedings, 4 July*, 2018; *Transcript of Proceedings, 13 August*, 2018, p. 4705). More broadly the industry was labelled “dishonest and greedy,” falling below community standards (Hayne, 2018, p. 73).

There was an exception, the Commission observed QSuper’s service was exemplary, calling them a “champion of Indigenous superannuation issues” (*Transcript of Proceedings, 13 August*, 2018, p. 4713). Lyn Melcer, QSuper’s Head of Technical Advice, with 40 years’ industry experience, testified firsthand observations of the barriers customers face to accessing services during a 2014 visit to the remote community of Lockhart River. Melcer also stated prior to 2014 she was not fully aware of the inequity such communities experience. This community experience drove Melcer to raise moral awareness through storytelling. Essentially moral awareness can, and in this case did, lead to taking ethical action, known as moral capacity (Schwartz, 2016).

This research asks how and why the moral awareness of EDM in Indigenous finance initially came to be manifested in QSuper, leading to the building of moral capacity. The role of moral awareness within EDM in finance is important; whilst much has been “discovered regarding the EDM process within business organisations, a great deal remains unknown,” particularly within this industry (Schwartz, 2016, p. 755; Trevino, 1986). There is a gap within the literature as to how finance organisations transition from a state of minimal awareness (potentially unethical, amoral, or immoral practice) to moral awareness leading to moral capacity which informs EDM to serve remote Indigenous customers.

Given the significance of the role of large financial organisations’ in the community, an appreciative case study into “what works” ethically has been explored (Hammond, 2013). The question posed in this interpretive, qualitative study was: “How and why does QSuper’s day-to-day decision-making deliver ethical finance services to Indigenous customers?” In this research EDM is considered as a function of both individual participant and contextual elements of QSuper (Ardichvili et al., 2009). The investigation operates within the premise that to understand what is going on ethically there needs to be moral awareness internally (Ardichvili et al., 2009; Meyers, 2004; Treviño et al., 1999). Key concepts of Indigenous methodology were applied such as ensuring the research was culturally safe, engagement with the process of decolonising research, and researcher accountability (Porsanger, 2004). The study sought to avoid relying solely on corporate reports because these are known to omit key elements of an organisation’s lived reality and this type of data holds elements of colonised functionalist conventions. To address this omission a “yarning” interview approach was adopted for the 29 participants which aligns to the interpretive, Indigenous approach of participants and researchers building a trusting relationship to co-construct the research reality (Bell, 2013; Bessarab & Ng'Andu, 2010).

The other sources triangulating in the analysis process were observational data and archival documents from the organisation as well as industry such as Commission transcripts and industry publications. There was considerable corroborating evidence between and within the various data sources of EDM practice incorporating storytelling, remote visits, and engagement in QSuper’s 100 + activities to do with improving Indigenous customer service which reinforced how empathy is lived throughout the organisation.

Practitioner contributions on shifting from minimal awareness, to raising moral awareness leading to growing moral capacity to strengthen EDM in Indigenous service in finance are offered. Practically demonstrating the lived experience of moral transition, which in QSuper’s case took six years, aims to decrease the gap between academia and real world application. Whilst moral awareness and moral capacity have been extensively discussed in the literature, it has not been linked to EDM in Indigenous finance incorporating storytelling in concert with empathy and inclusive practice. The contribution of storytelling to facilitate building moral capacity to deliver ethical services to Indigenous finance customers is new. The next section, a literature review is followed by the methodology, findings, implications, and then conclusion.

## Literature Review

Finance, in particular Indigenous finance, EDM, and research context are covered in this review.

### Finance

The purpose of finance in society is to facilitate economic flourishing through a mechanism of distributing goods and services which contributes to the common good (MacIntyre, 2007; Sison et al., 2019). Financial services organisations need to know their social purpose, their *telos* and how it is embedded at “both local and global, market, and non-market” levels (Asher & Wilcox, 2021; Moore, 2012, p. 7). Financial services organisations also need to be enablers of good practice where staff can “question existing cultures and practices with others who share similar concern” (Asher & Wilcox, 2021, p. 8). Reflective questioning of fiduciary duty and knowing the customers they are there to serve, is a challenge for large organisations given the variance in their customer’s needs. The process of financialisation has shut down some critical reflections on what constitutes an organisation’s social purpose as well as subordinates working within those organisations’ ability to question their existing culture and practices (Palley, 2008). A financial organisation wishing to provide ethical services must know their social purpose, their local market, and allow for questions to be asked about their culture. Applying a one size fits all approach to all Australian customers is not ethical—it does not demonstrate knowledge of the local market, nor does it fit the Australian Transaction Reports and Analysis Centre (AUSTRAC) criterion of knowing your customer (Austrac, 2020). Across the globe there has been increasing discussion on inclusive practice, which the industry refers to as financial inclusion (Kara et al., 2021). When there is a lack of inclusive practice, therefore little consideration of financial inclusion, it impacts “the level of financial innovation, poverty-levels, the stability of the financial sector, the state of the economy, financial literacy, and regulatory frameworks” (Ozili, 2021, p. 457). Indigenous, non-white, low literacy, and people with a disability are identified as being less likely to be included in mainstream financial institutions (Kara et al., 2021; Ozili, 2021).

#### Indigenous Finance

Financial disadvantage in collectivist Indigenous cultures impacts entire cohorts, not just an individual—this collectivist culture differs from an individualist Western financial management (Brimble & Blue, 2013; Lahn, 2012). Indigenous Australians have experienced “extreme financial disadvantage relative to other Australian citizens” (Breunig et al., 2019, p. 34). The flow-on effect from Indigenous economic inequality impacts housing, health, education, employment, imprisonment, and suicide rates (Coombes et al., 2018; Lowitja Institute, 2021). Whilst financial literacy is a longstanding, problematic issue in Indigenous communities, minimal research into mitigating risk exists. Commentators on Indigenous finance state organisational blindness prevails in financial organisations because directors believe “economic prosperity simply boils down to choice” (Bergsteiner & Avery, 2012; Pinto & Coulson, 2011, p. 75). Rationalising Indigenous financial disadvantage as a one-dimensional economic choice does not acknowledge the complexity of Indigenous barriers. Some academics have posited corporate reporting needs to extend beyond economic accountability to the triple bottom line: economic, social, and environmental accountability (Elkington, 1998; Pinto & Blue, 2016); others go further to a quadruple bottom line which includes cultural accountability reporting (Scrimgeour & Iremonger, 2004).

The finance industry in Australia, in particular superannuation service provision, is complex (Brimble & Blue, 2013). Various reports aimed at closing the gap between Indigenous and non-Indigenous people state literacy inequality is an ongoing issue—this is particularly problematic when customers are expected to use finance technology as the primary means to access financial services (Commonwealth of Australia, 2020; Ozili, 2021). Underlying education, structural, and cultural barriers contribute to individuals experiencing shame about financial capability. There is “a lack of confidence… embarrassment… and racism (real or perceived)” (Saunders & Piper, 2011, p. 7). To ignore these barriers is to ignore the purpose of finance, which is the distribution of the wealth of an economy for a good life to be shared amongst all customers within the community (MacIntyre, 2007; Sison et al., 2019). To mask these barriers, some organisations engage in blackwashing claiming they are committed to serving Indigenous communities whilst simultaneously rationalising not providing adequate, culturally appropriate services (Aston, 2022). The use of euphemistic language facilitates this rationalisation, stating “efforts for 3% of the population [Indigenous] does not fit into its ‘right sizing’ practice” (Cragg et al., 2009, p. 766).

Day-to-day behaviour in finance has been studied less than that in other industries; however, what is known is that finance staff hold functionalist assumptions where trust is placed in statistical instruments aligned to a positivist epistemology (Burrell & Morgan, 1979; Schinckus, 2015). Purely using statistical instruments for complex EDM does not allow for alternative narratives, including the stories of 3% of the population.

### Ethical Decision-Making in Financial Organisations

Whilst there are numerous approaches to EDM, in the context of exploring ethical practices in this case study, EDM pertains to a climate “where employees are not only expected to discern right from wrong, but go beyond this minimum to explore and implement ethical decisions when all choices seem right” (Ardichvili et al., 2009, p. 445). There are prescriptive approaches to EDM such as the contingency framework (Ferrell & Gresham, 1985) and moral intensity (Jones, 1991); however, these models do not go beyond the individual into a large complex financial organisation (O’Fallon & Butterfield, 2013). Various integrated EDM models have sought to display how moral awareness moves to moral capacity through feeling, sensing, reflecting, consulting, judging, and then committing to an action deemed right given the situation (Hannah et al., 2011; Schwartz, 2016; Trevino, 1986). These models also highlight when moral awareness is lacking, so too is moral capacity, thus EDM falls short. A lack of moral capacity may also indicate empathy is missing. Empathy is the ability to relate to another, which in turn raises moral action because ethical sensitivity increases, which simultaneously prevents ethical erosion (Brown et al., 2010; Yuguero et al., 2019).

There are elements of some EDM models which complement the ethical practice lens of this case because they approach EDM from a perspective of people within an organisation making decisions where there is a need for moral awareness (Loe et al., 2000; Schwartz, 2016). Day-to-day norms such as consultation and learning approaches need to be explored to ascertain how and why an organisation attains moral awareness (Schwartz, 2016). This is important because it is possible for moral dilemmas to be ignored or blocked by senior hierarchy, therefore, the culture is not one where staff have the scope to discern right from wrong (Ardichvili et al., 2009; Argyris, 2010).

Ethical decision-making involves the norms driving a process that recognises a moral dilemma, using some criteria or moral principles which leads to learning and action (Kohlberg, 1973; Schwartz, 2016). Unethical behaviour is known to take place when there is weak moral capacity, weak sanctions, as well as pressure from seniors and peers to engage in unethical behaviour (Schwartz, 2016).

To transition from moral awareness where concepts may be espoused (not lived) to moral capacity where morals are enacted (a lived reality), there is a process involving judgement, intention, and behaviour, as well as elements such as reward and punishment within the culture (Heyler et al., 2016; Treviño et al., 2006). Capturing assumptions, instead of settling for espoused cultural scripts is important to understanding how one relationship influences another in the EDM process (Schein & Schein, 2019). Studying the lived reality of Australian financial organisations is of particular importance because some moral issues have been overlooked since 1893 (Campbell, 1981; Hayne, 2019a; Hickson & Turner, 2002; Murray, 2014). More broadly investigating EDM in finance matters because it performs a “de-facto role as custodians of society’s resources” (Herbohn et al., 2019).

There are significant challenges to investigating large financial organisations because of their complexity: multiple realities need to be considered rather than simple functional economic rationalism (Schinckus, 2015; Waddock et al., 2016). Moral awareness is the first step in EDM (Rest, 1986). In this step, the realisation is made that the situation “requires a decision or action that could affect the interests, welfare, or expectations of oneself or others in a manner that may conflict with one or more moral standards” (Butterfield et al., 2000; Schwartz, 2016, p. 772). In situations where moral awareness is not present, the framing of an issue does not include moral terms, rather it is perceived in neutral or non-moral terms (Baucus & Rechner, 1995). Here perception precedes decision-making because “for the moral-decision process to begin, a person must recognise the moral issues” (Jones, 1991, p. 380). When there is a lack of moral awareness, that is, when people do not realise an ethical dilemma exists, there are either unintentional ethical consequences or unethical behaviour (Tenbrunsel & Smith‐Crowe, 2008).

In the process of EDM, leaders (sensegivers of organisations) use moral theories such as deontology to help inform their actions (Woiceshyn, 2011). These moral theories often have a truth-telling element (Hunt & Vitell, 1986). Truth-telling is linked to the basic moral principal of honesty. However, people working in finance have been found to have less honest norms than those in other industries (Cohn et al., 2014). Ethical storytelling has been used to convey honesty, it is the “truth-telling of the lived reality” for Indigenous governance which Elders state makes the world good and is not about “fairy tales” (Corntassel, 2009, p. 138; Driscoll & McKee, 2007). Indigenous Elders have used storytelling as an analytical ethical tool allowing for processes to be constructed, conveyed, and contested for millenniums. Whilst finance organisations often engage in storytelling to promote sales and myths about their founders, it is not known how storytelling is used to increase moral awareness and moral capacity in finance (Kemp et al., 2021). Nor is it known how storytelling could be used in an industry which generally identifies with positivist, functionalist characteristics (Morgan, 1988; Schinckus, 2015).

### Research Context

Two decades before the Commission, recommendations had been made to the finance industry on providing more equitable services to Indigenous customers through engaging in community-led activities, making long term commitments for change, increasing collaboration and partnerships for achieving cultural awareness, facilitating improvements to financial literacy, and financial inclusion; in short inclusive practice (Altman & Taylor, 2002; Banking Code Compliance Monitoring Committee, 2017). Whilst inclusive practice is a new concept for finance, health practitioners have considered the importance of inclusive practice for decades understanding a long term, people centred approach that is culturally focused, particularly for those who are hard to reach, is paramount (Bourke et al., 2021; Reserve Bank of Australia, 2021). The Commission found only 4% of branch buildings are located in regional and remote locations, yet 28% of Australians live there, which includes a number of Indigenous communities (Hayne, 2018). The Commission also found that branches in remote locations can still be inaccessible because of overly overcomplicated issues with performing a basic transaction. One case told to the Commission detailed a regional Indigenous finance customer’s journey to accessing a basic bank account with the help of a capable community worker. The customer needed to travel 3 h to visit the branch a number of times because the “banker embarked on a wide-ranging survey of the customer’s ‘needs’ evidently seeking to sell the customer other bank products” (Hayne, 2018, p. 260). Finance’s defence to the lack of remote branches is increasing electronic/fintech services and call centres. English is spoken in call centres—for some Indigenous customers English is their second or third language (Malcolm, 2011). If call centres are not able to respond to queries, customers are advised to visit a branch (Hayne, 2018).

Finance organisations have not integrated the industry’s Indigenous guidelines or lived up to their espoused values (Hayne, 2019a). The Commission detailed financial advisers had been incentivised to use high-pressure tactics to sell finance products to vulnerable communities, enhancing these organisations’ short-term profit (Hayne, 2018). In effect, staff have been systematically rewarded for failing to uphold their fiduciary duty (Magnan & Martin, 2018). The industry’s behaviour has a sizeable impact on vulnerable people, because it can cause economic loss, which in turn has a negative impact on individuals and communities: psychologically, socially, and physically (Hayne, 2019a, 2019b). As a whole, the Australian finance industry is one of the least trusted (Endelman, 2022); only 20% of people believe banks are ethical and just 21% believe financial services organisation have their best interest at heart (Punt, 2018). The next section details how complex EDM in finance in this case study has been explored.

## Methodology

An interpretive case study is a good fit for exploring how and why QSuper deliver ethical financial services to Indigenous Australian customers. Numerous design options were considered to investigate the phenomena before opting for a single case study involving three data sets (yarning-interviews, archival documents, and observations), to enable particularisations to be identified (Weick, 2007; Yin, 2018). This methodology incorporates Indigenous epistemology to “make visible what is special and needed, what is meaningful and logical in respect of Indigenous peoples” (Porsanger, 2004, p. 107). A storytelling (“yarning”) approach was adopted for the interview process; and within the individual interview process multiple forms of yarning conversations occurred (Kvale, 2007). The somewhat relaxed yarning approach differed from the more formal narrative inquiry to encourage “a journey both the researcher and the participant share as they build a relationship and visit topics of interest to the research” (Bessarab & Ng'Andu, 2010; Geia et al., 2013, p. 15).

When the researcher met a participant there was initially “social yarning,”an informal and unstructured conversation to build trust so participants were then comfortable to share knowing that “the researcher is accountable to the research participant” (Bessarab & Ng'Andu, 2010, p. 40). This was important at the time of interview and all throughout the research process (Gibson et al., 2020). During the unstructured social yarning time, topics included COVID-19 adjustments, news, and shared knowledge about Indigenous communities in different parts of Australia. Once the participant was ready, the dialogue switched to “research topic yarning”: semi-structured interviews, “through participant stories that are related to the research topic” (Bessarab & Ng'Andu, 2010, p. 40).

Reflexivity was a multi-faceted process throughout the case study (Lucas et al., 2021). From the outset there was transparent self-reflectivity. The majority of the research team are not Indigenous including the primary interviewer who was raised in a Western-context which could create distance because ontological Western thinking can be destructive to Indigenous knowledges (Martin, 2017; Nicholls, 2009). Non-Indigenous researchers discussing first-person positioning with Indigenous researchers was helpful, particularly on appropriate language use. Interpersonal reflectivity was also important. For example, during social yarning it was important to be aware of people at the base of the organisational hierarchy could feel their voice was of less importance; therefore, to actively promote a psychologically safe space (Porsanger, 2004). Interpersonal reflectivity also facilitated staying on topic. In one instance a researcher and participant connected on a shared concern for people accessing hostels (accommodation for people who would otherwise be sleeping on the streets) during COVID-19 lockdowns (Nicholls, 2009). Without reflectivity social yarning could have circumvented topic yarning. Reflexive practice when writing up the post-interview notes detailing elements of the social yarning was also beneficial to the researcher’s sensemaking of the issues.

Collective reflexivity took place at multiple points (Ripamonti et al., 2016). For example, at the end of topic-related yarning, to facilitate the interviewer accurately capturing the participants voice, there was a circling back on key participant points to ensure their most salient arguments were captured and allow space for clarification. Collective reflectivity also occurred when working through the analysis between the researchers. In several instances these discussions led to recursive action between the analysis phases and to what initially appeared as a theme, later with more reflection, was a concept. One other element of collective reflexivity was the researchers going back to QSuper with findings and discussing themes with them on multiple occasions.

A purposive approach was used to engage QSuper because it met the criterion of a large financial organisation which was reported by the Commission to be delivering Indigenous services ethically (Lewis, 1985; *Transcript of Proceedings, 13 August*, 2018). Established in 1913, initially to support Queensland Government staff, this superannuation, insurance, and investment organisation is now open to anyone. In February 2022 QSuper merged with Sunsuper, the newly formed not-for-profit organisation, Australian Retirement Trust (ART), has over 2 million customers with more than $230 billion funds under management (Australian Retirement Trust, 2022).

Twenty-nine participants (16 men and 13 women), from all levels of the QSuper hierarchy were engaged in research topic yarning (semi-structured interviews). Each interview lasted an hour on average, resulting in 1616 pages (290,663 words) of transcripts. Two participants identified as Indigenous. The yarnings were designed to draw out participants’ day-to-day lived experience (Weick, 2017); therefore the majority of interviews (71%) were conducted face-to-face in the participants’ work environment. The other interviews (29%) were conducted over the phone because of distance and COVID-19 restrictions. A requisite variety of data generated 50–90 initial codes from each yarning engagement (Bencherki et al., 2019).

Observation notes and archival documents were triangulated with the yarning data (Polkinghorne, 2005; Tracy, 2010). Archival documents included reports and transcripts from the Commission, industry publications, QSuper annual reports, as well as QSuper documents given to the researchers from the participants. These QSuper documents included emails from Indigenous Elders of the communities they had visited, internal e-news communications with staff, as well as documents QSuper provided to the Commission (Hayne, 2018; Indigenous Superannuation Working Group, 2019). Of note was a document listing 101 of QSuper’s activities to improve and facilitate its Indigenous customer service, a version of this document was presented to the Commission. Activities included participation at Indigenous working groups, QSuper staff mentoring young Indigenous entrepreneurs, projects with different regulators, commissioning local Indigenous artists, and cultural training—it also listed engagement with 78 external organisations.

The 101 activities document was helpful in corroborating evidence with other data sources. For example, in one yarning session, participant E42 discussed commissioned Indigenous artwork, “We have created bespoke artwork using local artists for each of our offices across the state to provide a more welcoming environment and to educate our staff and clients.” When the researcher entered the building and one of the meeting rooms they saw local “Gilimbaa,” artwork as described by E42 and detailed in the 101 activities document. Further corroboration of this particular artwork and the artist’s organisation was its display in some of QSuper’s corporate reports.

To ascertain themes from the data a four phase thematic content analysis was conducted which encompassed coding (I), categorising (II), conceptualising (III), and deriving themes (IV) (Braun & Clarke, 2006; Clarke & Braun, 2018; Cunliffe, 2003; Saldaña, 2015). The benefit of this four phase manual analysis was that it enabled the identification of underlying assumptions, rather than settling for a collection of content domain summaries (Holloway & Todres, 2003; Lincoln & Guba, 1985; Nowell et al., 2017). Initially coding of five yarning scripts revealed some categories, the most dominant being “customers are at the heart of all we do,” which ultimately ended being a key concept. During this time there was some triangulation with the other data sources to gain confidence as to whether some categories would go forth to become a concept based on corroborating evidence. Yarning-interviews continued until data saturation was reached (Fusch & Ness, 2015).

Once all the transcripts were coded there was further triangulation with the other data sources. Key categories were detailed into excel spreadsheets, concepts were then identified through the use of notetaking, mind mapping, listening and re-listening to the yarning, and reflective discussions on the keyness of concepts, not just volume, to establish what concepts were corroborated or contradicted (Braun & Clarke, 2006; Tee et al., 2014). Whilst time intensive, this manual coding was important for sensemaking and ensuring latent themes were not missed (Clarke & Braun, 2018). As mentioned earlier, the ongoing reflexive process called for recursive action across the four phases as data codes and themes were re-read, discussed multiple times, notes expanded upon, mind maps redrawn, transcripts re-read, and reflected upon until interpretive awareness was reached (Cunliffe, 2003; Nicholls, 2009).

## Findings

Key themes to emerge from the QSuper data on EDM for Indigenous customers were storytelling, empathy, and inclusive practice. How the organisation engaged in these behaviours day-to-day gradually shifted them from minimal awareness of the need for increased focused on EDM when serving Indigenous customers to moral awareness, then moral capacity over approximately six years. Participants were quick to point out that whilst progress had been made, “It’s a learning process, we’ll learn something new next week” (E51). The next sections unpack each of the key themes which have brought the organisation to a state of moral capacity.

### Storytelling

#### Story Telling Leading to Moral Awareness

Melcer’s 2014 Lockhart River visit has become part of the organisation’s story: a number of participants referred to it during their interview, “Lyn initially coming out to Indigenous communities with Australian Securities and Investment Commission (ASIC), it means we, Indigenous people, work more with serving our customers” (F49). Externally, Melcer’s visit has featured in industry publications, as well as the Commission hearing (Braddon, 2017; *Transcript of Proceedings, 13 August*, 2018). When Melcer told her story to the Commission, she shared there was initially a low moral awareness of the barriers Indigenous customers experience:

…having difficulties meeting our rules on identification. Driver’s licenses don’t exist, passports don’t exist… I thought we treated all our customers equally because we had exactly the same rules for everyone. What Lockhart River showed me is not everyone starts in the same place… We assume everyone has a driver’s license but they don’t (*Transcript of Proceedings, 13 August*, 2018, p. 4714).

In 2014 when Melcer shared her story with the QSuper board the exchange created a new moral awareness. This awareness became the “first interpretative step of the ethical decision-making process,” in improving Indigenous customer service for QSuper (Brown & Treviño, 2006, p. 602). So impactful was the storytelling a former CEO said: “Lyn, we need to do more of this. You tell me what you want” (E50). Melcer credits some of the board’s emotion and rationalisation, which led to action (moral capacity), to the principle that “with human stories, there’s no way you can *not* want to help.” Evidence of the efficacy of storytelling impacting moral capacity years later was apparent in the yarnings of six QSuper directors, who gave endorsement for ethical Indigenous customer service. One referred to the work of improving Indigenous services as what they have been most proud of during their board tenure, “I’m particularly happy with the work we do with Indigenous communities, connecting and engaging them with their super” (D27). Other directors spoke of participating in remote visits (D29) and sponsoring Indigenous entrepreneurs (D30).

Melcer’s storytelling spread throughout the organisation**.** Participants spoke of Melcer’s passion for vulnerable customers: “Lyn’s got this wonderful trustee spirit, she also knows a lot and because of the values she legitimately puts our customers first holistically…It means people [staff] are more consistent and courageous with our principles” (E33). One element that made Melcer’s storytelling impactful was her technical background: “Lyn is known as a technical expert, the technicalities she knows is just incredible—she gets the importance of relationship building too” (D26). Technical finance practitioners are generally highly analytic in their methods and do not normally opt for storytelling; however, Melcer believed the Indigenous service issue was of such moral significance that this approach was imperative. Melcer was explicit in setting the tone for the storytelling: human-centred, where vulnerable people must not be blamed or seen as transactions. This focus on right language use is consistent with how Melcer behaves day-to-day. Other participants corroborated how Melcer’s human-design approach flowed throughout QSuper, differentiating it from other financial organisation’s processes because “We use customers’ names,” (F44) rather than transaction numbers.

#### Service Access

Participant stories of Indigenous service access incorporated known and unknown barriers. Known barriers participants wanted to see addressed directly and indirectly included “racism” (E52), “transport” (E50), and “literacy” (F49). One barrier participants said is increasingly overlooked in other organisations such as government, health, and banking, is the “utilitarian one size fits all approach” (E50). Utilitarianism is problematic for superannuation organisations attempting to ethically serve remote communities because they are reliant on other institutions to complete elements of their claim form. For example, a medical review is needed to complete an early release claim form; however, if a remote customer cannot access this service it may mean they do not receive their funds before they die.

Another barrier story participants spoke of which is not covered in the academic literature was non-conventional forms of identification (ID). On remote visits, QSuper staff assist anyone wanting to access their superannuation regardless of the financial organisation holding the funds. Participants assisting community members from other finance organisations told stories of the other finance organisations not being aware of, or adopting, new AUSTRAC (Australian Transaction Reports and Analysis Centre) legislation on non-conventional forms of ID (AML/CTF Rules) which meant vulnerable people could not access their funds (Austrac, 2020). Other organisations had inaccurately advised Indigenous community members saying: “We can’t do that because we’re breaking the law,” the participant added, “It’s not breaking rules, we’ve got this ability to use flexibility [AML/CTF Rules]” (E51).

#### Trust

Participants told stories of the importance of being continuously trustworthy with communities, “They will be watching you as soon as you land” (E51) and then the need to follow through on their word when working with Indigenous customers and associations. Melcer said: “One of the things that we do that I’ve heard others don’t do is, we always follow through.” The consequence of not following through Melcer explained was “it breaks the trust of the community,” and the organisation would not be welcomed again. One benefit of being trustworthy with a community is they tell positive stories about QSuper to other communities: “You could not have paid for how well these people were promoting us [to another remote community] we didn’t have to say anything” (E51). This action of follow through was not dissimilar to the concept of governance which emerged strongly in the analysis. Detailed processes, reminiscent of government processes, supported staff’s adherence to compliance. Directors and middle managers spoke of products and processes which were required to be trustworthy. Participants were aware of the need to be a trusted person, “A fiduciary, acting in the best interest for another person” (E37). Stories were plentiful of supporting customers experiencing “financial hardship,” (D28) and, or uniting people with their “deceased loved one’s superannuation accounts” (E45). There was considerable pride and a sense of purpose in helping hardworking, “loyal public servants” (F49) achieve their long-term financial goals. The next sub sections in the findings feature more exerpts from participant’s stories.

### Empathy

#### Vulnerable Customers are at the Heart of What we do

Throughout the data there was overarching corroboration of “customers being at the heart of what we do” (D30); that QSuper people were serving fellow Queenslanders—the word Queensland being used 80 times by the 29 participants. This customer centric assumption incorporated vulnerable customers. Empathy for the customer was embedded throughout the organisation, its origin deriving from the organisation’s government beginnings. Now, as a non-government business the empathic value is driven by directors through role modelling, recruitment, and resource allocation.

Two questions asked throughout the organisation fostering ongoing empathy are: (a) “What is the right thing to do by the customer?” (E48) and (b) “What if this was my brother, mother, father, sister?” (E51). These questions have deep moral implications which help guide individual staff as well as teams and the broader organisation in day-to-day EDM. Melcer spoke of how the questions helped her and her team navigate change internally and externally, “The number of times I hear me say ‘what’s the right thing to do?’” Similarly, directors stated when making decisions on policies, programmes, and products, they referred to: “What is the right thing to do by the customer?” (D25). The CEO stated that asking the question was the rationalisation for “sponsoring DV [domestic violence] Connect, that has major benefits for our police and ambulance customers” (D25). Other participants referred to how the two questions provide ethical guidance in their daily work because, “That’s in the best interest of the customer—having the customer heart” (E36). There was a concern for the long-term financial welfare of the customer, that today’s service will benefit people in “10, 20, and 30 years’ time” (D28) as well as considering how the decisions or advice will impact customers “kids and grandkids” (F46).

All participants wanted to, and many felt, they “make a difference” (E31) in their customer’s lives. There was also a strong awareness they were “looking after other people’s money” (E39); therefore, they needed to avoid unnecessary spending on advertising, workplace facilities, technology, and travel. The importance of recruiting customer centric, trustworthy staff was mentioned by many participants, as was extensive vetting in onboarding to ensure there was a proper cultural fit. Reinforcing this “fit” (D28) was the balanced scorecard remuneration (REM) approach which incorporates living the organisational values.

Customer empathy was also expressed in the way participants explained superannuation is complex: “it’s not a topic most people understand and there is no word for it in Indigenous language” (E51). Another expression of this empathy was ensuring every customer, “even difficult customers are listened to and respected” (F44). High levels of service were expected whether the customer was a “judge, member of parliament, nurse, or police officer,” (E50), “retiree struggling to pay the rent, or a widower of a deceased customer” (E46).

#### Empathy Differentiates QSuper

Most participants spoke of how QSuper was different from the “big four” (E37) banks and other finance organisations. QSuper’s empathy logically led participants to “keep customer fees low,” (D30) which in turn led to why their REM assumptions differed from industry—for why REM bonuses were a “lower reward” (E36) than others. Some advocated for the complete elimination of bonuses, “I don’t see a lot of other professions getting bonuses” (E34). Similarly participants spoke of the way QSuper views and reviews how they can pay an insurance claim “even if it’s not better for us [short-term] it’s better for the customer,” (E45) whereas other organisations focus on avoiding claim payment: “working for one of the big four the bottom line was more important than the customer” (E45).

Customer empathy at QSuper was a lived reality compared to an individual revenue focused business model at other finance organisations. F42 explained: “The conversations [in previous organisation] were around how much money did *you* make this week? Never around how did you help someone.” F42 went on to say “QSuper’s ethical focus is what attracted me. I left working in a bank because I wanted to be somewhere more aligned with my ethics.” In addition to the formal vetting processes to facilitate recruiting candidates with empathetic values, there was informal behaviour to protect the moral integrity of the organisation. Some participants told stories of protecting the culture through actively discouraging unethically behaving former colleagues from applying for a job at QSuper because they were: “Too focused on themselves, on how much money they make, not on the people on the street who are our customers. There’s lots of really hard-nosed people, not empathetic” (E38). E38 relayed a story of being disgusted a past colleague did not face any disciplinary action for writing “Mrs Smith agrees to $7000 in fees [where signature should be],” in their previous workplace (E38). A number of participants said they did not want past colleagues who forge customers’ signatures and engage in fee gouging to bring such behaviour into QSuper.

#### Practical Action: Signs of Moral Capacity

Between 2014 and 2020, QSuper moved from one person having moral awareness of the barriers Indigenous customers experience to decreasing barriers and proactively engaging with communities, in the process building internal moral capacity in this area. Practically, one of the first behaviour changes was a focus on five postcodes which have a high Indigenous population: “We proactively reviewed customer postcodes in Far North Queensland looking at unclaimed money, lost accounts,  and deceased accounts in those areas” (E50) (QSuper, 2020). QSuper then contacted customers, or loved ones of the deceased, to reunite them with their superannuation, in time becoming more efficient with the assistance of the Australian Tax Office (ATO). Knowledge gained from providing better Indigenous services was shared at Indigenous finance summits, as well as with regulators, and when assisting other institutions to conduct remote visits (Indigenous Superannuation Working Group, 2019). Part of QSuper’s sharing at these forums involved emphasising the importance of consultation and collaboration with Elders, Indigenous businesses and associations as well as others involved in service access and delivery. One example of a collaboration was the development of a Memorandum of Understanding with Queensland’s Registry of Births, Deaths, and Marriages to facilitate identification. Melcer said: “People who have got issues with identification, have issues with financial literacy, it stops our customers being able to access their money.” QSuper also addressed vulnerable customer ID issues through its relationships with legislators and regulators, such as AUSTRAC and ASIC. Melcer was part of a working group that facilitated a review of the *Anti-Money Laundering and Counter-Terrorism Financing Rules Instrument Act 2006,* which now recognises non-conventional forms of ID for vulnerable community members (AML/CTF Rules).

#### Learning—Decreasing Stigma

Indigenous participant F49, spoke of complexity in the call centre and how deep listening is needed to avoid stereotyping customers. There is a risk, F49 explained, of perceiving a customer as unintelligent because English is not their first language:

Don't assume that they’re dumb because they’re not. They’re very intelligent people. If you go into a conversation with an Indigenous person, with respect of their history, their culture, and who they are, you will achieve a lot together. From my experience, you need to spend time to understand the challenges faced in our remote communities around accessing basic services, such as a phone, the internet, it’s so different” (F49).

F49 shared that call centre people had benefitted from Indigenous learnings, which provided insights into better servicing Indigenous customers, as well as other vulnerable customers, such as new migrants and victims of domestic violence.

At the time of interviewing, an increasing number of QSuper teams had started to acknowledge Traditional Custodians at the beginning of their meetings. In the insurance area Indigenous learning has been incorporated into the business coaches’ remit. Business coach F45 relayed a case of an Indigenous customer accessing their superannuation before they died and that without the improved tailored service, the customer would not have been have achieved this:

We changed our procedure for him [customer] because he was financially illiterate, he could not fill in forms. Our outreach person worked with the social worker to interview the doctor so we could pay his claim. This is what you need to be doing, not treat people as though one size fits all. (F45).

A further element of this case involved prompting the customer to identify a beneficiary of their finances, because they were about to die without explicit instructions (a valid will). Once the customer’s case was closed, the coach (F45) and frontline staff managing the case shared with their team how they addressed the initial problem and worked to resolve the issues, as part of their approach to ongoing learning.

### Inclusive Practice

#### Range of Activities

A key element of how QSuper developed moral awareness for ethically serving Indigenous customers was through inclusive practice. This involved staff working side by side with Indigenous people, rather than the top-down traditional Western approach of we know what is best for you (Porsanger, 2004). Middle manager E52 spoke of the importance of actively seeking local Indigenous insight because community differences can be substantial. Before a visit to Thursday Island, an Elder highlighted QSuper’s proposed customer information packs had land-based artwork, which could cause offence because “It doesn’t have any water references, which is Torres Strait” (E52). E52 described this as “a good lesson of engaging appropriately before going into a community.”

The commencement of QSuper’s moral awareness journey would not have started if Melcer had not been open to engaging in inclusive practice on a remote community visit. One of the norms of Melcer’s day-to-day work is consulting internally as well as externally. Evidence to corroborate these norms included participants making comments such as, “Lyn got on the Indigenous superannuation working group” (E51), as well as articles in industry reports. Directors and staff now participate in inclusive practice which takes a range of forms—many of these engagements are detailed in the 101 activities document such as working alongside Indigenous finance counsellors to serve customers and develop culturally sensitive learning resources (QSuper, 2020).

#### Know Your Customer

One element of inclusive practice that differentiated QSuper from others within the industry was understanding the importance of “knowing their customer,” which includes life expectancy. In remote communities the average life expectancy can vary significantly from the Australian population average of 81 years for men and 83 for women (Australian Institute of Health & Welfare, 2021). The average life expectancy for Indigenous men in some remote locations, such as Cape York, Queensland is 58 years which falls below the standard superannuation industry release date of 60 years (Barker, 2019). Melcer alluded to the lack of inclusive practice regarding age of access as amoral and a prohibitor to Indigenous customers engaging in finance organisations saying: “They go ‘Look I really can’t be bothered with super because I’m probably never going to get to retirement age and I don’t understand it.’” The scenario of the dying customer detailed earlier is another example of the importance of knowing your customer.

#### Remote Visits

QSuper’s visits to remote communities is not a functionalist, discrete activity, rather, it involves extensive remote community consultation and the inclusion of organisations such as the Indigenous Consumer Assistance Network, ATO, Centrelink, and Good Shepherd Micro Finance. The learning which takes place on remote visits is highly impactful for driving staff’s “focus and passion” (D29). Staff learn what roles people perform in the community, which assists them later with problem solving on non-conventional identification. Barriers remote customers face become very real to staff when they too are faced with not being able to access their phone “up there [remote community] Optus [telecommunications provider] doesn’t work, you may be able to use one other telco but they charge you for it, so that’s not fair” (E51). One director relaying their remote visit said, “It certainly throws up the challenges in how we service remote and disadvantaged work—there is more to do” (D29). Part of the inclusive practice incorporates reflective learning. Evidence of QSuper engaging in reflection came from stories as well as a document covering common questions and answers from remote visits—this working document is also shared as a resource Indigenous financial counsellors draw upon in their day-to-day work.

### Synthesis of Findings

**Fig. 1:**
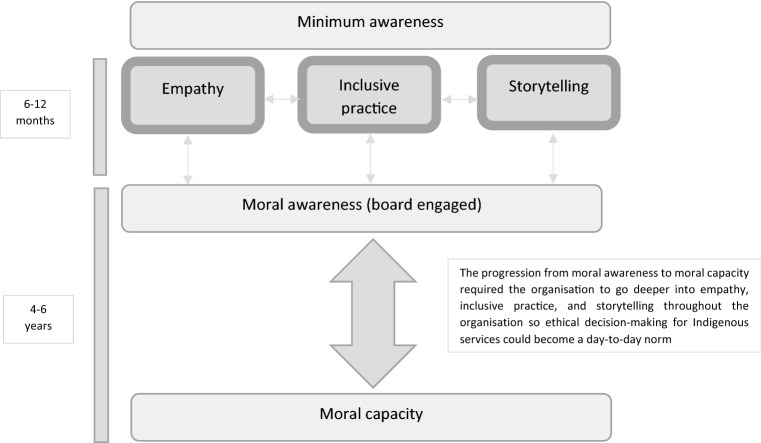
Enhancing moral capacity for Indigenous services in large finance organisations

Figure 1 details a synthesis of the findings. That is, how the large finance organisation shifted from minimal awareness to moral awareness and eventually moral capacity. Three core interacting factors help to inform and enhance the other: empathy, inclusive practice, and storytelling. Initial moral awareness occurred in a relatively short period of time with the directors’ support: 6–12 months, when the three factors were enacted. For sustainable organisational change enactment of the three factors needed a longer time frame of 4–6 years to build moral capacity. The text box in the figure, emphasises this point: the three factors which brought about moral awareness are also what brings about moral capacity when they are sustained and embedded throughout the organisation. For example, an inclusive practice experience such as listening to an Indigenous customer on a remote visit and asking what the right thing to do is, then engaging in one off storytelling will raise moral awareness initially. However, it is the ongoing asking of what the right thing is to do throughout the organisation, continually engaging with Indigenous communities, and the normalising of storytelling from all staff which builds moral capacity. Having the three factors role modelled by directors facilitated the behavioural engagement; that is, the tone from the top meant it was right for staff to feel empathy for Indigenous customers and make time to participate in activities where they will hear and tell stories to ethically serve Indigenous customers. In this case study moral capacity means EDM for Indigenous customers is not an activity policy makers engage in once a year, rather, it is a day-to-day consideration for all people in the organisation hierarchy.

## Implications

### Practitioner Implications

Key implications for practitioners wanting to improve EDM in Indigenous customer service are customer centricity, incorporating the vulnerable, empathy, and visits to remote communities. These were approaches QSuper took, which saw them move from minimal awareness to moral awareness, and then moral capacity. Melcer was keen to stress there were no cost or low costs to the organisation for these changes: “We haven’t hired any staff, this hasn’t cost us anything.” One example of the no cost approach was embedding customer centric values to ensure it included vulnerable customers. This financial inclusion-customer centric value was actively embedded by directors. There was an awareness one size does not fit all, which extended to taking action (moral capacity), ongoing learning and altering processes to serve different customers (Schwartz, 2016). “We share stories”(Melcer); was another no cost way QSuper sought to address barriers, facilitate truth telling, and solve problems.

Prior to 2014, recognition of vulnerable customers was part of QSuper’s culture; however, it did not fully extend to recognising the barriers Indigenous customers encounter. Effective storytelling heightened moral awareness of Indigenous customers which fitted into the organisation’s existing values and knowing their customers. This awareness is indispensable in some communities where life expectancy is 58 years, and the standard preservation date for many superannuation funds is 60 years (Barker, 2019). Organisations who do to not know their customers risk denying them access to their funds whilst they are living.

Implicit in QSuper’s customer centric value is trust. Several participants spoke of the importance of keeping their word: “Staff say they follow-up and they always do”(D27)—we “follow our principles” (E39). If an organisation failed to keep their word it is unlikely they will be trusted to come back to community, because the storytelling between communities would be negative. QSuper staff’s empathetic ethic connection with Indigenous communities resulted in them being highly recommended to other communities.

Proactively engaging with Indigenous customers in remote areas provided learning, which cannot be taught from reading a document in a city building; it also offered scope for internal storytelling. Having internal creditable technical staff leading the storytelling, rather than a sleek PR campaign, was beneficial for engaging the emotion and judgement of staff in a functionalist business area.

The ongoing empathic questioning of “What is the right thing to do by the customer?” and “How would I feel if this was my mother/family?” creates fertile soil for moral awareness. Participants shared that the need for financial inclusion involved ongoing learning to receive: “education from Elders and the community” (E52) and other Indigenous associations. E52, who had worked on a number of QSuper’s Indigenous initiatives, emphasised the need for a long term approach explaining the development of moral capacity was not achieved through a one-off training activity, rather, a concerted integrated effort over multiple years. Storytelling, empathy, and remote visits will assist financial practitioners wanting to make a lasting, positive legacy in their organisation: “because nothing replaces meeting and talking to people, visiting people firsthand—it is so powerful” says Melcer. Initially making a small step to create moral awareness, can lead to moral capacity in staff, connecting them with the organisation’s ultimate telos, whilst simultaneously making a big change in Indigenous customers accessing their finances. These approaches could also be applied to other large organisations, such as telecommunications and energy providers to improve their ethical delivery to Indigenous customers, as well as other vulnerable groups.

### Academic Implications

This study fills a gap in the literature previously unexplored: storytelling within finance organisations to improve EDM in Indigenous service. Within this case study, storytelling did not sit in isolation, rather it was integrated with empathy and inclusive financial practice. A key advantage of storytelling is its engagement with the left hemisphere of the brain for everyday language tasks, as well as the right hemisphere, for comprehending complex tasks (Jung-Beeman, 2005). Superannuation products are complex. Engaging in process changes for a complex superannuation product requires bringing together complex cognitive processes (neurological microcircuitry) with the logical everyday language function (emotional–imaginative) (Jung-Beeman, 2005). This is why storytelling can assist learning in finance. Indeed storytelling played a critical role in shifting QSuper, and could move other finance organisations, from low levels of awareness to higher levels of moral awareness and then moral capacity when serving Indigenous customers.

The integrated EDM approach posited here directs organisations to listen and engage with customers Indigenous economic principles and morals, as well as longstanding issues such as financial literacy, physical access to services, and cultural recognition (Peterson & Taylor, 2003, p. 105). Inclusive practice stands in stark contrast to a Westernised decision-making approach where there is little scope for storytelling that may reveal lived experiences of organisations preventing vulnerable customers accessing finance. What differentiates this study from the abundance of case studies on inclusive practice is the other literature is mostly situated in a healthcare setting—there is minimal literature detailing how empathy, inclusive practice, and storytelling is beneficial for EDM in finance (Allen et al., 2020).

The empathetic contribution: questioning the right thing to do and alternating the word customer for mother, challenges opportunistic finance actions, characteristic of “organisations as being machines made up of parts and the purpose of organisations as being solely profit-making activities” (Pavlovich & Krahnke, 2012, p. 136). Organisations engaged in day-to-day empathic questioning cannot operate in moral blindness when it comes to vulnerable Indigenous customers, because they would soon hear Indigenous stories. As implied throughout the case study, the whole of the organisation was engaged, so too the proposed contribution posits moral awareness throughout the different parts of the organisation needs to be interconnected so the entity as a whole can connect to the community it serves. Empathy creates connection between the parts of an organisation and the communities they serve, which contributes to a society becoming united (Waddock, 2009).

The three integrated factors in this finance context are posited as an academic contribution. *Empathy* initially drove Melcer to engage in *inclusive practice*, that led to *storytelling*, which then led to moral awareness and eventually moral capacity. Prior to 2014 QSuper’s functionalist industry knowledge had not supplied the organisation with sufficient impetus to engage in EDM in Indigenous customer service at a deep level. It was not until staff started engaging in inclusive practice, listening to people’s stories, that a more comprehensive understanding of vulnerable customers’ reality occurred (O'Connor, 2009; Shahid et al., 2019). Whilst this inclusive practice is new to finance, Indigenous communities have known the benefits of such learning practices for millennia (O'Connor, 2009).

The Western positivist paradigm normalised in finance where profit maximisation is the primary value was found to be incompatible with EDM to serve Indigenous customers. QSuper differed from the norm through its embedded vulnerable customer value which calls them to be trusted stewards for people they esteem as family. This empathic value serves as an organising mechanism to create a more humanistic and caring work environment; it also has the capacity to dissolve the Western power-distance between self and others (Pavlovich & Krahnke, 2012). Participants spoke of the significance of community visits: “The stories we [QSuper] get from serving, the impact its having on our staff who are coming back and telling those stories—its having an effect” (E52). This “effect” on EDM when integrated with the day-to-day work be it investment, policy development, or call centre work appears to be creating a balance between: “social good and self-interest, trust-based and legal relationships, teamwork and individual achievement, risk taking and caution, business and society, locally sensitive and foreign” (Chen & Miller, 2010, p. 22).

In essence QSuper’s evolving EDM process involving three factors is some of the “why” QSuper were recognised as being different from other finance organisations in the Commission. The normalised sharing of real world finance stories of Indigenous customers combined with asking what the right thing is to do (?) does not allow them to continue to operate at the same standard as everyone else if the standard is questionably unethical. The moral behaviour of QSuper working in different departments, performing different roles, has not come about by chance. Rather through an intentional engagement with storytelling, empathy, and inclusive practice, to continually build moral capacity.

## Conclusion

The exploratory, interpretive analysis into QSuper’s EDM when serving Indigenous customers posits integrating storytelling, empathy, and inclusive finance practice contributes to increasing day-to-day EDM leading to increased moral awareness and eventually moral capability. Whilst EDM in finance has been explored before (Boatright, 2013; Prentice, 2007), it has not incorporated the role storytelling plays for shifting an organisation from minimal awareness to moral capacity when providing ethical services to Indigenous customers. It is acknowledged storytelling has been explored as beneficial to improving ethics in industries such as education, healthcare, and the arts; however, it has not been discussed in literature relating to financial EDM (Driscoll & McKee, 2007; Rieger et al., 2018). Two questions which tilled the soil of the case study’s culture to facilitate inclusive practice and normalise storytelling were: “What is the right thing to do by the customer?” and “How would I feel if this was my mother/family?” The Commission and other investigations found financial organisations contribute to Indigenous customers’ disadvantage through decision-making which fosters systematic, exploitative practices, and significant unintended consequences (Hayne, 2019a; Saunders & Piper, 2011).

QSuper was recognised in the Commission as different: their moral awareness was apparent from the six years of inclusive practice which led to building moral capacity. Through storytelling, a QSuper technical manager engaged directors’ emotions and then others, to lead their organisation to judgement and action. Since 2014, directors have delivered congruent messages, internally and externally, on the need to increase Indigenous financial capability because this aligns with their enacted (vulnerable) customer value. This value has propelled QSuper to advocate for improved regulations for identification and increased financial capacity nationally. Internally QSuper adjusted its policies, products, and services impacting Indigenous customers and other vulnerable groups.

Whilst QSuper engages with many Indigenous stakeholders, this study was limited to staff. One limitation of this case was only two participants identified as Indigenous. It is recommended that future studies incorporate a broader scope to capture more Indigenous voices. A similar case study to this one could also be carried out on another large organisation in a different industry, such as telecommunications or energy, to identify corroborating or contradictory EDM themes and barriers. Another acknowledgement is the design of the study follows Indigenous methodology and is appreciative regarding EDM on Indigenous service. This research does not assert all QSuper activity (now ART) undergoes EDM. The reasoning behind the design was to explore what works and to “do no harm” to the participating organisation (Hugman et al., 2011, p. 1271). Few financial organisations in the post-Commission climate are open to such enquiry. QSuper’s opening to external consultation, an action many organisations refer to in their corporate reports, is not in reality frequently engaged in at a meaningful level. This lack of integrity is a disservice to many of the 490,000 finance staff who seek to make a positive difference in their customer’s life yet are denied the opportunity to ethically serve through barriers which can be broken down through moral awareness.
